# Risk of Childhood Overweight after Exposure to Tobacco Smoking in Prenatal and Early Postnatal Life

**DOI:** 10.1371/journal.pone.0109184

**Published:** 2014-10-13

**Authors:** Susanne Eifer Møller, Teresa Adeltoft Ajslev, Camilla Schou Andersen, Christine Dalgård, Thorkild I. A. Sørensen

**Affiliations:** 1 Institute of Public Health, Epidemiology, Biostatistics and Biodemography, University of Southern Denmark, Odense, Denmark; 2 Institute of Preventive Medicine, Bispebjerg and Frederiksberg Hospitals, The Capital Region, Denmark; 3 Institute of Public Health, Environmental Medicine, University of Southern Denmark, Odense, Denmark; 4 Novo Nordisk Foundation Centre for Basic Metabolic Research, Faculty of Health and Medical Sciences, University of Copenhagen, Copenhagen, Denmark; Johns Hopkins Bloomberg School of Public Health, United States of America

## Abstract

**Objective:**

To investigate the association between exposure to mothers smoking during prenatal and early postnatal life and risk of overweight at age 7 years, while taking birth weight into account.

**Methods:**

From the Danish National Birth Cohort a total of 32,747 families were identified with available information on maternal smoking status in child's pre- and postnatal life and child's birth weight, and weight and height at age 7 years. Outcome was overweight according to the International Obesity Task Force gender and age specific body mass index. Smoking exposure was categorized into four groups: no exposure (n = 25,076); exposure only during pregnancy (n = 3,343); exposure only postnatally (n = 140); and exposure during pregnancy and postnatally (n = 4,188). Risk of overweight according to smoking status as well as dose-response relationships were estimated by crude and adjusted odds ratios using logistic regression models.

**Results:**

Exposure to smoking only during pregnancy, or both during pregnancy and postnatally were both significantly associated with overweight at 7 years of age (OR: 1.31, 95% CI: 1.15–1.48, and OR: 1.76, 95% CI: 1.58–1.97, respectively). Analyses excluding children with low birth weight (<2,500 gram) revealed similar results. A significant prenatal dose-response relationship was found. Per one additional cigarette smoked per day an increase in risk of overweight was observed (OR: 1.02, 95% CI: 1.01–1.03). When adjusting for quantity of smoking during pregnancy, prolonged exposure after birth further increased the risk of later overweight in the children (OR 1.28, 95% CI:1.09–1.50) compared with exposure only in the prenatal period.

**Conclusions:**

Mother's perinatal smoking increased child's OR of overweight at age 7 years irrespective of birth weight, and with higher OR if exposed both during pregnancy and in early postnatal life. Clear dose-response relationships were observed, which emphasizes the need for prevention of any tobacco exposure of infants.

## Introduction

Worldwide, the prevalence of overweight children and adults has increased greatly over the past three decades [1]–[3]. Although recent research suggests a levelling off in the obesity epidemic, childhood overweight remains an important health problem [4], [5].

Smoking during pregnancy is associated with overweight in offspring, which has been demonstrated in a number of studies and confirmed by recent meta-analyses [6]–[10]. Even though Oken et al's meta-analysis confirmed the association, the analysis was limited by small studies, different definitions of overweight, and by heterogeneity in offspring ages [8]. Maternal smoking during pregnancy may cause intrauterine growth restriction, and is associated with low birth weight (BW) [11]. Limited numbers of studies have looked into the influence of low BW [12] and catch up growth in early childhood on this [13]–[15]. Catch-up growth refers to accelerated growth in early infancy to compensate for low BW [11], [16], [17], and has been demonstrated to be an independent risk factor for childhood overweight [9], [18]. Other known risk factors are also likely to modify the association between smoking and childhood overweight, such as maternal obesity, maternal gestational weight gain, socio-occupational status, and breastfeeding [7], [9], [19]. Few studies demonstrate dose-response relationship between maternal smoking during pregnancy and risk of overweight in childhood [10], [20]–[22]. A recent study by Harris et al. revealed that exposure to parental smoking during pregnancy increased the risk of overweight in daughters in adolescence and adulthood in a dose-response manner [23]. Although this was investigated in a very large sample, information on exposure was based on long term recall and limited to exposure of smoking during pregnancy [23].

The child's early postnatal period, while being breastfed, is suggested to be a critical period for mothers smoking exposure [24]. Therefore, we suspected that exposure to maternal smoking also in the early postnatal period throughout lactation might prolong the negative effect on child's health, and may in turn increase the risk of childhood overweight. However, previous studies of the postnatal period are few and the results are not conclusive [24]–[26]. In a birth cohort study with follow-up at 8 years of 609 children, Florath et al [25] showed that smoking by both mothers and fathers pre- as well as postnatally were associated with increased BMI of their children at 8 years of age. However, they interpreted their findings as indications of confounding by life style, rather than specific intrauterine effects of maternal smoking, which clearly calls for more investigations.

With use of a large cohort, including comprehensive information on mother and child, the objectives were to investigate dose-response relationships of mother's smoking during the child's prenatal period. Furthermore, the aim was to investigate whether mother's prolongation of smoking into the child's early postnatal life while breastfeeding the child, was an independent risk factor, in an otherwise healthy population of mothers and children. Finally, we wanted to investigate whether these relations were independent of the child's BW.

## Materials and Methods

Data from The Danish National Birth Cohort (DNBC) were used, which originally included 101,042 pregnant women, who gave birth to a child between 1996 and 2002 [27]. Mothers were interviewed by telephone twice during pregnancy, approximately at week 16 and 30 (Interviews 1 and 2) as well as postnatally, when the child was 6 and 18 month old (Interviews 3 and 4). The 7-year follow-up was carried out from 2005 to 2010 using a mailed questionnaire, where 53,888 mothers and children responded. Mothers were asked to report their child's latest measurement on height and weight at the 7-year follow-up [28], [29].

**Figure 1:**
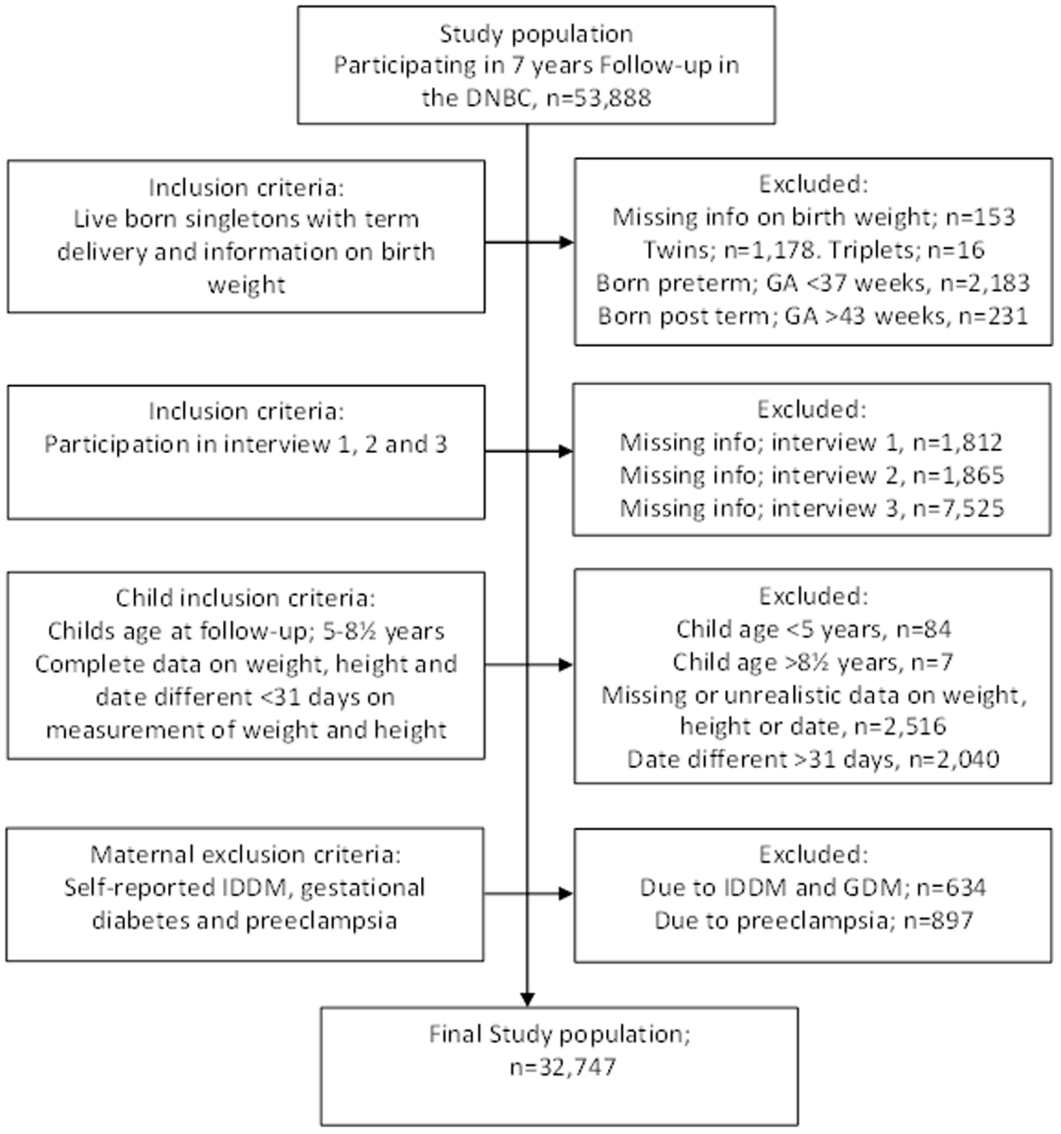
The figure show a flow chart of the study population.

To be included in the present study, the children should be born singleton, alive, and at term (≥37 gestational weeks). Only mother-child pairs, where mothers had participated in Interviews 1, 2 and 3, and with children's complete data on height and weight at the 7-year follow-up were included. Also, child's height and weight should have been measured less than 31 days apart, and the child's age should be between 5 and 8½ year at follow-up. In 31 children, height and weight measures were changed to missing, due to possible errors in the registered measures; being height below 100 cm or above 150 cm, or weight below 15 or above 45 kilos, corresponding to the criteria set by the DNBC, www.dnbc.dk. We excluded mothers with preeclampsia, gestational diabetes and diabetes mellitus. A total of 32,747 mother-child pairs fulfilled these criteria. The inclusions and exclusions are shown in Figure 1.

### Ethical statement

The Danish Committee on Biomedical Research Ethics has approved the Danish National Birth Cohort (case no. (KF) 01-471/94). Each participant gave written informed consent at enrollment into the Danish National Birth Cohort. According to the principles stated by the ethics comitees, participants have the right to have their data removed from the cohort at any time. Children born into the cohort participate on their mothers written informed consent until they are able to decide for themselves at age 18 years. The Danish Data Protection Agency has approved the cohort (case no. 2008-54-0431) and the 7-year follow-up (case no. 2004-41-4078). The Danish Data Protection Agency and the Institutional Board Committee of the Danish National Birth Cohort approved the present study.

### Exposure variable

**Figure 2:**
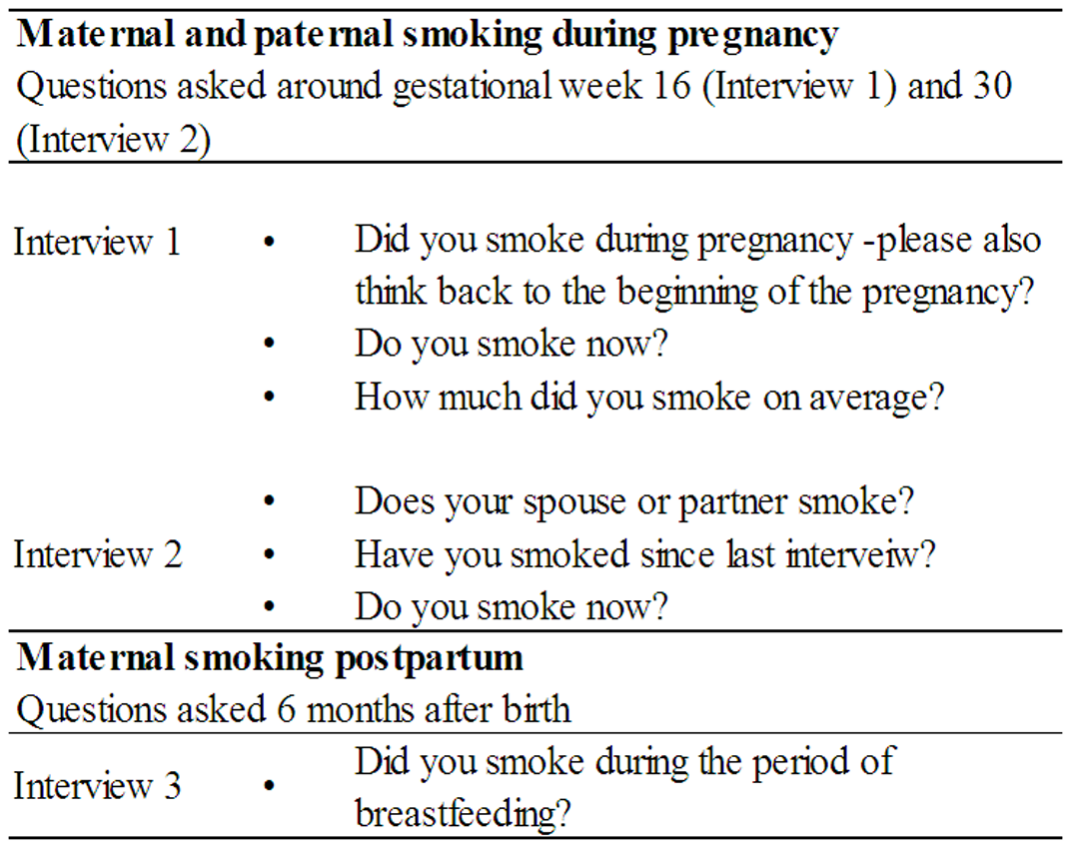
The figure show questions assessing information on maternal and paternal smoking exposure from Interview 1 (obtained around gestational week 16), Interview 2 (obtained around gestational week 32), and Interview 3 (obtained 6 months after child birth).

Maternal smoking was assessed both at Interview 1 and 2 during pregnancy. Postnatal smoking habits from birth through 6 months during the period of lactation were assessed in Interview 3. The questions assessing maternal smoking are shown in Figure 2. The questions could be answered with yes or no, except the question concerning quantity of smoking during pregnancy. If a woman answered yes in either Interview 1 or in Interview 2, she was categorized as a smoker during pregnancy. The study population was categorized in four groups according to answers from Interviews 1 to 3∶1) No smoking 76.6% (n = 25,076); 2) Smoking only during pregnancy 10.2% (n = 3,343); 3) Smoking only postnatally 0.4% (n = 140); and 4) Smoking both during pregnancy and postnatally 12.8% (n = 4,188). Information on quantity of maternal smoking in numbers of cigarettes smoked daily in early pregnancy was obtained from Interview 1, Figure 2. We used quantity of maternal smoking as a continuous variable (1-54 cigarettes) and subsequently categorized it into six groups; 0, <1, 1–4, 5–9, 10–14, 15–19 and ≥20 cigarettes daily, where 0 cigarettes daily were non-smokers.

### Outcome variable

The endpoint was overweight at age 7 years. Body Mass Index (BMI = weight (kg)/height (m) ^2^) was calculated based on the recorded weight and height at the 7-year follow-up. We used the International Obesity Task Force standards to define overweight in children with age- and sex specific cut off points for BMI [30]. Overweight was recorded as a dichotomous variable with obese children included in the overweight group. The reported data on the children's height and weight have been validated previously [31], which showed that they were without any noteworthy random or systematic bias.

### Covariates

From Interview 1, information on parity, socio-occupational status, paternal smoking, as well as maternal pre-pregnancy weight and height was obtained. Parity was coded as either primiparous or multiparous. Socio-occupational status was categorized as high, middle, and low, based on the parents combined status. High represented manager position or long/middle long education, middle represented skilled workers or workers with short education, and low represented unskilled workers, with no education, or on special allowance. The question about paternal smoking was coded either smoking or non-smoking, and it was included as a possible confounder influencing the child's odds ratio of overweight independently of the mother's smoking [25], [32], [33]. Using self-reported pre-pregnancy weight and height, maternal pre-pregnancy BMI was calculated and used as a continuous variable and subsequently categorized in groups for descriptive purpose; <18.5 kg/m^2^,18.5–24.9 kg/m^2^, 25–29.9 kg/m^2^ and ≥30 kg/m^2^. From Interview 3, information on maternal gestational weight gain and exclusive breastfeeding was recorded. Both gestational weight gain and breastfeeding were used as continuous variables and categorized into groups; <10 kg, 10–17.99 kg and ≥18 kg and 0 (no breastfeeding), <14 weeks, 14–22 weeks and >22 weeks, respectively. Paternal BMI was calculated from height and weight measures reported at Interview 4, and used as a continuous as well as group variable; <18.5 kg/m^2^,18.5–24.9 kg/m^2^, 25–29.9 kg/m^2^ and ≥30 kg/m. From The Danish Medical Birth Registry information on pregnancy outcome, maternal age, date of birth, gestational age at birth, child sex and BW was obtained. Gestational age was used as a continuous variable. We categorized BW into groups; <2,500 gram, 2,500–4,000 gram and>4,000 g, with low BW defined as a BW <2,500 gram.

### Statistical analyses

Between-group differences were tested with Chi-square tests. Risk of overweight, if exposed to maternal smoking during pre- and postnatal life, was estimated by crude and adjusted odds ratios (OR) using univariate and multiple logistic regression analysis with adjustments according to two models, Model 1 and Model 2. Exposure to smoking during and/or after pregnancy was categorized into the described four groups with no smoking as the reference group. We tested for interactions in the association under study and with the following covariates; maternal pre-pregnancy BMI, BW, breastfeeding and child sex. We found no interactions with any of these covariates, except for BW. Thus, maternal pre-pregnancy BMI, and child sex were included as covariates in Model 1 adjusted analyses and breastfeeding was included as a variable contributing to the associations in Model 2 analyses. As low BW modified the effect of maternal smoking on child's risk of overweight additional analyses stratified on BW with adjustment for confounding according to Model 1 were performed. In the first adjusted Model 1, the following pre-specified covariates were entered: Maternal age, maternal pre-pregnancy BMI, gestational weight gain, paternal smoking, socio-occupational status, parity, gestational age, child sex and BW. Model 2 included Model 1 covariates and paternal BMI and breastfeeding. Dose-response relationships were investigated in various ways through multiple logistic regression models to estimate the risk of overweight with increasing quantity of smoking. Analyses were performed for different groups corresponding to either smoke exposure during pregnancy only, or smoke exposure both during pregnancy and postnatally adjusting for prenatal smoke amount. Quantity of maternal smoking was included in groups as described previously, and adjusted for confounding according to Model 1. Moreover, as no interaction with smoking postnatally (yes/no) was observed on risk of overweight with prenatal smoking, another logistic regression model was performed, which included prenatal smoking as a linear variable and smoking postnatally as a categorial variable. In all analyses, a p-value<0.05 was considered statistically significant. Since some mothers (n = 1,233, 4,1%) were entered twice into the cohort, additional regression analyses, using a cluster option taking into account the dependency between the siblings, were carried out for the main results; however, as expected, this did not alter the estimates and widened the confidence intervals only minimally, so the results are presented without this correction. Statistical analyses were performed using: STATA release 11 IC software.

## Results

### Smoking habits

A total of 76.6% were nonsmokers and 23.4% reported smoking during pregnancy and/or postnatally, Table 1. Mothers who smoked during pregnancy had higher maternal gestational weight gain and were of lower socio-occupational status. They gave birth to children with lower BW and were more likely not breastfed or had “short duration” of breastfeeding than children of nonsmoking mothers (all p-values<0.001), Table 1.

**Table 1:** Distribution of covariates in relation to maternal smoking status.

Maternal smoking		No smoking		Smoking only during pregnancy		Smoking only postnatally		Smoking during pregnancy and postnatally		
		n = 25,076		n = 3,343		n = 140		n = 4,188		
		%	±SD	%	±SD	%	±SD	%	±SD	p-value*
		76.6		10.2		0.4		12.8		
**Birth weight (g)**	Mean	3.688	±486	3.658	±507	3.714	±507	3.456	±494	
	<2,500	0.5		1.1		0.7		1.9		<0.001
	2,500–4,000	73.0		73.5		72.2		83.6		
	>4,000	26.5		25.4		27.1		14.5		
**Parity**	Primiparous	44.2		57.4		37.9		42.6		<0.001
	Multiparous	55.8		42.6		62.1		57.4		
**Sex**	Boy	51.6		50.0		49.3		49.5		0.027
	Girl	48.4		50.0		50.7		50.5		
**Breast feeding (weeks)**	Mean	16.8	±6.8	15.2	±7.7	17.6	±5.7	14.4	±7.3	
	None	4.0		8.3		4.3		3.4		<0.001
	<14	17.1		22.6		9.4		34.1		
	14–22	63.4		56.5		72.6		52.9		
	>22	15.5		12.6		13.7		9.6		
**Maternal age (years)**	Mean	30.9	±4.1	30.0	±4.2	30.1	±3.7	30.5	±4.6	
	16–25	6.0		11.2		7.2		11.3		<0.001
	25–29.99	38.0		41.7		45.0		35.7		
	30–34.99	39.8		34.9		36.4		35.8		
	≥35	16.2		12.2		11.4		17.2		
**Socio-occupational status**	High	73.6		66.9		77.2		51.7		<0.001
	Middel	24.2		29.7		22.1		41.5		
	Low	2.2		3.4		0.7		6.8		
**Maternal gestational Weight gain (kg)**	Mean	14.7	±5.1	17.3	±6.3	16.8	±6.3	15.1	±6.2	
	<10	12.2		7.7		7.9		15.9		<0.001
	10–17.99	61.9		47.0		55.4		51.6		
	≥18	25.9		45.3		36.7		32.5		
**Maternal pre-pregnancy BMI (kg/m^2^)**	Mean	23.4	±4.0	23.3	±4.1	22.8	±3.1	23.3	±4.1	
	<18.5	3.8		4.5		6.4		6.3		<0.001
	18.5–24.9	70.8		70.4		69.8		67.7		
	25–29.9	18.5		18.5		20.9		18.6		
	≥30	6.8		6.5		2.9		7.4		
**Paternal BMI (kg/m^2^)**	Mean	25.0	±3.7	25.3	±3.7	25.8	±4.1	25.3	±3.4	
	<18.5	0.4		0.2		0.0		0.3		<0.001
	18.5–24.9	55.5		52.1		54.2		52.8		
	25–29.9	38.1		40.3		35.0		38.2		
	≥30	6.0		7.4		10.8		8.7		
**Paternal smoking**	No	79.6		55.1		59.3		38.3		<0.001
	Yes	20.4		44.9		40.7		61.7		

Abbreviations: BMI: Body Mass Index. Missing information on variables; Parity (n = 16), maternal gestational weight gain (n = 284), maternal pre- pregnancy BMI (n = 453), socio-occupational status (n = 91), paternal smoking (n = 500), paternal BMI (n = 6,570) and breastfeeding (n = 8,998). * χ^2^-test.

### Overweight at 7 years of age

A total of 9.4% (n = 3,085) of the children were overweight. Overweight was more prevalent in girls (10.4%) than in boys (8.5%) (p<0.001), Table 2. Overweight children had higher BW and were more likely not breastfed or had “short duration” of breastfeeding. Their parents were more often overweight or obese and more often of lower socio-occupational status (all p-values <0.001, except for maternal age p = 0.027), Table 2.

**Table 2:** Distribution of covariates according to normal weight and overweight children.

		Normal weight		Overweight*		
		n = 29,662		n = 3,085		
		%	±SD	%	±SD	p-value**
**Child weight group at age 7 years**		90.6		9.4		
**Birth weight (g)**	Mean	3.643	±490	3.782	±518	
	<2,500	0.8		0.5		<0.001
	2,500–4,000	75.4		65.3		
	>4,000	23.9		34.2		
**Parity**	Primiparous	45.7		41.2		<0.001
	Multiparous	54.3		58.8		
**Sex**	Boy	51.7		46.3		<0.001
	Girl	48.3		53.7		
**Breast feeding (weeks)**	None	4.1		6.8		<0.001
	<14	19.2		24.2		
	14–22	62.0		56.8		
	>22	14.7		12.2		
**Maternal age (years)**	Mean	30.7	±4.1	30.7	±4.3	
	<25	7.1		8.5		0.027
	25–29.99	38.2		37.3		
	30–34.99	38.9		37.9		
	≥35	15.8		16.3		
**Socio- occupational status**	High	71.2		60.0		<0.001
	Middel	26.0		35.7		
	Low	2.8		4.3		
**Maternal gestational weight gain (kg)**	Mean	15.0	±5.3	15.2	±6.4	
	<10	11.8		15.9		<0.001
	10–17.99	59.9		51.2		
	≥18	28.3		32.9		
**Maternal pre- pregnancy BMI (kg/m^2^)**	Mean	23.2	±3.9	25.6	±5.0	
	<18.5	4.5		1.6		<0.001
	18.5–24.9	72.2		52.6		
	25–29.9	17.4		29.5		
	≥30	5.9		16.3		
**Paternal BMI (kg/m^2^)**	Mean	24.9	±3.1	26.6	±3.7	
	<18.5	0.4		0.0		<0.001
	18.5–24.9	56.6		37.2		
	25–29.9	37.3		48.1		
	≥30	5.7		14.7		
**Paternal smoking**	No	72.7		63.9		<0.001
	Yes	27.3		36.1		

Abbreviations: BMI: Body Mass Index. Missing information on variables; Parity (n = 16), maternal gestational weight gain (n = 284), maternal pre-pregnancy BMI (n = 453), socio-occupational status (n = 91), paternal smoking (n = 500), paternal BMI (n = 6,570) and breastfeeding (n = 8,998). *Obese children are included in the overweight group. ** χ^2^-test.

### Smoking habits and risk of overweight

**Figure 3:**
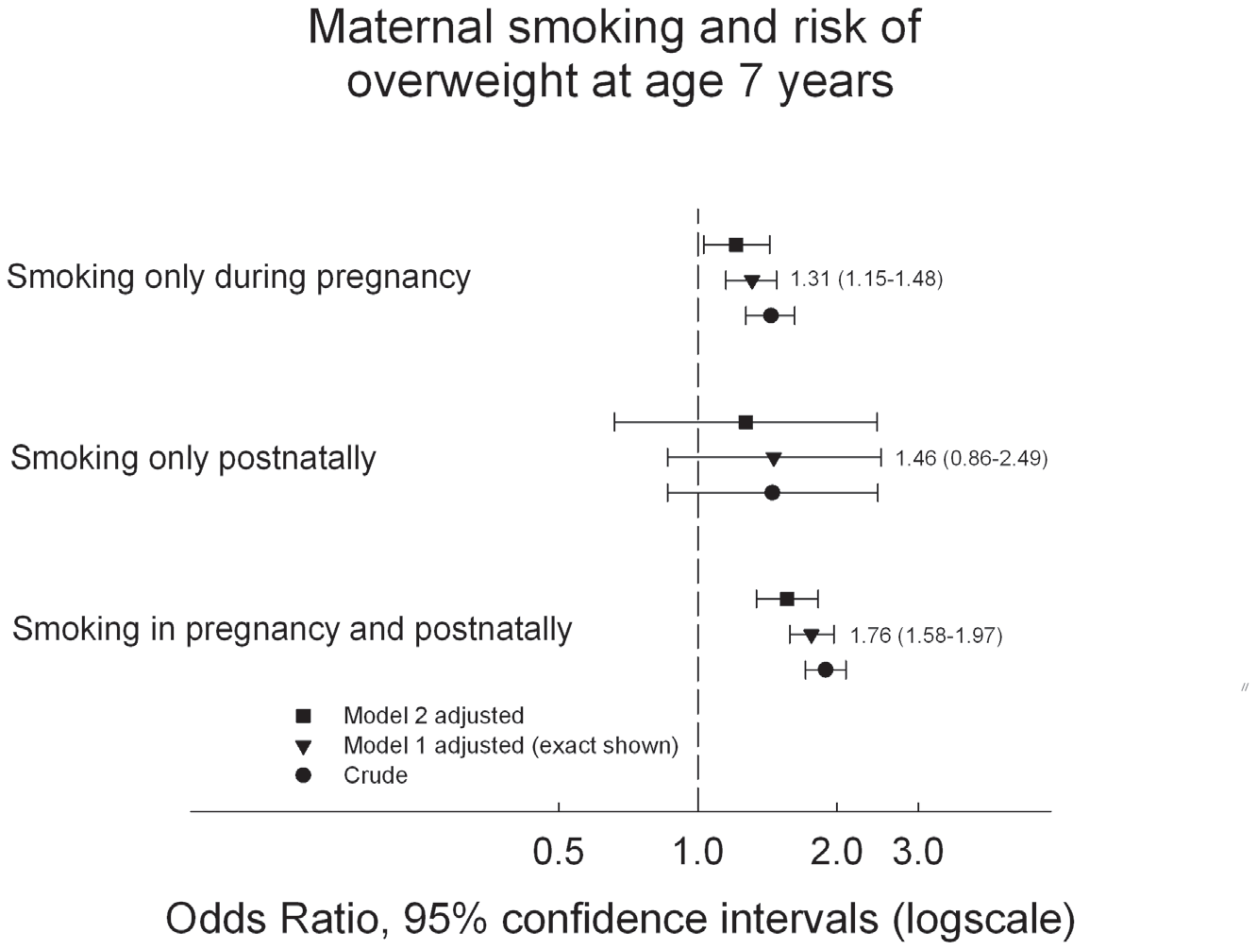
The figure shows the odds ratios with 95% confidence intervals of overweight at age 7 years by different exposure windows of maternal smoking pre- and postnatally. The reference group is children of non-smoking mothers. Model 1 analyses are adjusted for child sex, parity, birth weight, maternal gestational weight gain, maternal age, maternal pre-pregnancy BMI, paternal smoking and socio-occupational status. Exacts estimates with Model 1 adjustments are shown in parentes. Model 2 is adjusted for the same covariates as Model 1, but with additional adjustment for paternal BMI and breastfeeding. Obese children are included in the overweight group.

Figure 3 shows the risk of overweight at age 7 years with different exposure windows during pregnancy and postnatally. Exposure to smoking only during pregnancy or both during pregnancy and postnatally were significantly associated with childhood overweight at age 7 years (OR: 1.31, 95% CI: 1.15–1.48, and OR: 1.76, 95% CI: 1.58–1.97, respectively). A similar tendency was found for the association between exposure to smoking only postnatally and childhood overweight (OR: 1.46, 95% CI: 0.86–2.49). In addition, a significantly higher risk of overweight was observed with exposure to smoking both during pregnancy and postnatally than exposure to smoking only during pregnancy (OR: 1.35, 95% CI: 1.16–1.56). All results were adjusted according to Model 1. None of the included confounders altered the result notably when tested separately, except adjustment for paternal smoking which slightly lowered the risk estimates. In the adjusted model, paternal smoking increased the OR of overweight (OR: 1.27, 95% CI: 1.17–1.39). Additional adjustment (Model 2) for paternal BMI and breastfeeding influenced the risk estimates to a minor degree, Figure 3. Thus, only Model 1 adjustments were carried forward in analyses of pre- and postnatal smoking in which we investigated and adjusted for prenatal quantity of smoking.

### Quantity of daily smoking and risk of overweight

**Figure 4:**
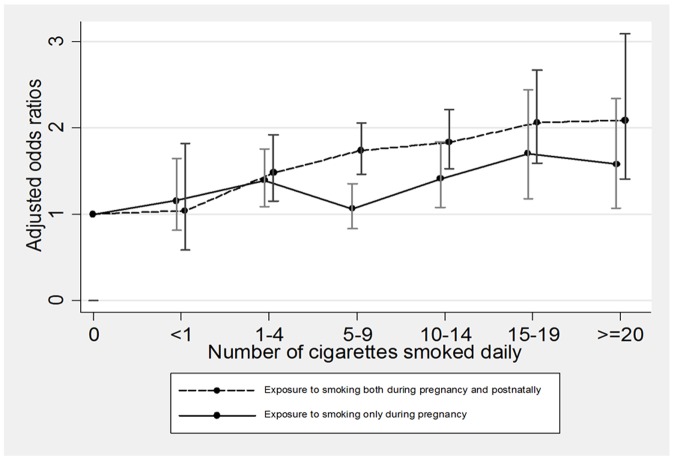
The figure shows the odds ratios of overweight at age 7 years with increasing numbers of cigarettes smoked daily during pregnancy by two different exposure periods, either exposure only during pregnancy, or exposure both during pregnancy and postnatally. The odds ratios were adjusted for child sex, parity, birth weight, gestational age at birth, maternal age at birth, maternal gestational weight gain, maternal pre-pregnancy BMI, paternal smoking and socio-occupational status.

Increasing number of cigarettes smoked daily during pregnancy was significantly associated with increased risk of childhood overweight at age 7 years. This pattern appeared for groups of smoking only during pregnancy, as well as smoking both during pregnancy and postnatally, Figure 4. The prenatal dose-response relationship was significant; per one additional cigarette smoked per day an increase in risk of childhood overweight was observed (OR:1.02, 95% CI: 1.01–1.03), Table 3. The risk of overweight increased significantly if exposed to smoking both during pregnancy and in early postnatal life than to exposure only during pregnancy (OR:1.28, 95% CI: 1.09–1.50), Table 3.

**Table 3:** The table shows the odds ratios (OR) with 95% confidence intervals (CI) of overweight at age 7 years in children exposed to smoking both during pregnancy and postnatally compared with children exposed only during pregnancy, while adjusting for quantity of prenatal smoking.

Overweight*		Adjusted OR	95% CI	p-value
**Smoking during pregnancy and postnatally**	vs. smoking exclusively in pregnancy	1.28	1.09–1.50	0.002
**One cigarette increase/day during pregnancy**		1.02	1.01–1.03	0.003
**Maternal age (years)**		1.02	0.99–1.03	0.117
**Maternal gestational weight gain (kg)**		1.02	1.01–1.04	<0.001
**Maternal pre- pregnancy BMI (kg/m^2^)**		1.12	1.11–1.14	<0.001
**Paternal smoking**	vs. no paternal smoking	1.38	1.18–1.60	<0.001

Odds ratios were additionally adjusted for postnatal smoking, child sex, parity, birth weight, gestational age at birth, and socio-occupational status (not shown). Only mother-child pairs, with information on numbers of cigarettes smoked daily during pregnancy, as well as with information on either smoking during pregnancy only, or smoking both in pregnancy and postnatal, are included in these analyses (n = 6,804). *Obese children are included in the overweight group.

Odds ratios of included covariates i.e. maternal age at birth, maternal gestational weight gain, maternal pre-pregnancy BMI, and paternal smoking are shown as well.

### Smoking habits, birth weight and risk of overweight

The prevalence of children born with BW <2,500 gram was 0.8% (n = 246) in the study population. Low BW was more frequent in children of smoking than non-smoking mothers. Within the small group of children born with low BW, the risk of overweight was considerably increased if exposed to maternal smoking during pregnancy (Crude OR: 12.60, 95% CI: 2.40–65.55) than in low BW children not exposed to smoking during pregnancy. Hence, adjusted analyses showed that children with BW≥2,500 gram, who were exposed to maternal smoking also had a significantly higher risk of overweight at 7 years of age compared with the unexposed children. This was the case if exposed to maternal smoking only during pregnancy as well as both during pregnancy and postnatally (OR: 1.29, 95% CI: 1.13–1.46 and OR: 1.76, 95% CI: 1.57–1.96, respectively) than in children of non-smoking mothers.

## Discussion

This large prospective cohort study showed an increased risk of overweight in children at age 7 years, if exposed to maternal smoking during pregnancy or both during pregnancy and in early postnatal life compared with children of non-smoking mothers. In addition, a clear dose-response relationship was observed between quantity of prenatal smoking exposure and risk of overweight at age 7 years, while taking into account postnatal smoke exposure. Moreover, exposure to smoking both during pregnancy and postnatally lead to a significantly higher risk of overweight compared with smoking exposure only during pregnancy. Thus, maternal smoking either during pregnancy, or postnatally are both independent risk factors for overweight at age 7 years and is independent of BW and breastfeeding.

A growing number of studies have reported maternal smoking during pregnancy as a risk factor for childhood overweight [8], [10], [19], [34]–[37]. In 2008, Oken et al conducted a meta-analysis on the association between maternal smoking during pregnancy and offspring's risk of overweight, and confirmed that maternal smoking during pregnancy was significantly associated with childhood overweight (OR: 1.50, 95% CI: 1.36–1.65) [8]. The meta-analysis was based on 14 observational studies including in total 84,563 children. However, none of the individual observational studies consisted of more than 8,000 participants, and the meta-analysis was weakened by different definitions of overweight and by heterogenerity in offspring ages (ranging from 3 to 33 years) [8]. The present study, which used a uniform definition of overweight assessed at a well-defined age in a very large population of 32,747 mother-child dyads, could confirm the results from the meta-analysis. Also, we were able to show a very clear dose-response relationship, ie, increasing numbers of cigarettes smoked daily during pregnancy increased the risk of overweight in childhood. This is in agreement with results from another recent study which found a similar dose-response pattern [23]. We were able to expand these findings, by demonstrating that prolonged exposure to smoking both during pregnancy and continuing into the early postnatal period, while adjusting for smoking intensity during pregnancy, further increased the risk of overweight.

We found that low BW modified the association between maternal smoking during pregnancy and risk of overweight at 7 years of age. Indeed, children born at term with low BW had a much higher risk of childhood overweight if they were exposed to maternal smoking during pregnancy than children with low BW not exposed to smoking during pregnancy. This may either be a chance finding or have a yet unknown biological basis, such as a particular susceptibility in low birth weight infants to smoking-induced maternal inflammatory oxidative stress, leading to epigenetic changes and/or programming effects in the child, directly or indirectly related to fat storage and metabolism [38]. When excluding the children born with low BW from the analyses, the risk estimates remained largely unchanged. Thus, our results suggest that children with normal BW (≥2,500 gram) also have an increased risk of overweight if exposed to maternal smoking during pregnancy, revealing that the risk of overweight is independent of low BW, but may be mediated through rapid infant growth. This finding is in agreement with a recent study by Beyerlein et al showing that among 12,383 German children, low BW was unlikely to be the main cause for the association between intrauterine smoking exposure and higher BMI in later life [12]. Adjustment for paternal smoking weakend the strength of the association and father's smoking may be an independent risk factor. In a recent study Florath et al. showed that paternal smoking and smoking of both parents at pre- and postnatal periods increased risk of offspring overweight [25]. Future studies should assess the influence of both maternal and paternal smoking exposures, in which the timing and the possible effects of passive smoking is investigated in more detail both before pregnancy, during pregnancy and postpartum.

Our study sought to address the independent influence of exposure to maternal smoking during the postnatal period only, on risk of overweight. However, the group with exposure to smoking only postnatally was small (n = 140). We found a tendency towards an increased risk of overweight if exposed to maternal smoking only postnatally, but it was not statistically significant. The observation was nevertheless in agreement with a previous study by Raum et al, who found a positive association between exposure to maternal smoking in the child's first year and childhood overweight (OR: 2.08, 95% CI: 1.02–4.24) [24], but others have failed to detect such an association [26]. On the other hand, our study found an increased risk of overweight in children exposed to smoking both during pregnancy and in early postnatal life compared to exposure only during pregnancy, after controlling for the quantity of smoking during pregnancy, which suggest that exposure postnatally is also an important risk factor. Maternal smoking during lactation establish a route for direct exposure to tobacco components. Hence, if mothers smoke and breastfeed then smoking may impose an even larger effect on the child's health, and may in turn increase the risk of childhood overweight. In contrast, breastfeeding is generally found to have a protective effect on overweight and may therefore be beneficial in spite of the possible transmission of chemicals through the breastmilk [7]. Breastfeeding may act both as an effect-modifier and a mediator of the association between postpartum smoke exposure and overweight and the influence may depend on the timing of smoking while breastfeeding and whether the mother inhale the nicotine. Our study found no interaction with mother's breastfeeding, and inclusion of breastfeeding in our categorical analyses of pre-postpartum smoke exposure suggested marginal influence from breastfeeding on the association at interest. Future studies should address the complex influence of mothers smoking while breastfeeding by investigating all beneficial and negative effects. One parameter to measure is the nicotine content of the breastmilk while obtaining information on when mothers smoke while breastfeeding, such as just before or after she breastfeed the child.

In contrast to the conclusions about the observed associations being due to confounding by other life style factors drawn by Florath et al [25], we suggest that the contention that exposure of the fetus and the infant to the chemical content of smoke is a likely cause of the later overweight may be justified. Moreover, the finding of an even further increased risk of overweight in children who were also exposed to mother's postpartum smoking, and the findings of an increased risk of overweight if fathers smoke as well, suggest that any direct or passive smoking exposure of infants in public or private areas should be avoided, also to avoid other adverse effects [39].

Strength of this study was the prospectively collected information especially on smoking in a large general population based birth cohort which gave us exceptional good opportunities to study the association without the risk of recall bias. However, some limitations have to be kept in mind. Exposure to smoking was based on self-reported smoking habits obtained by interviewer-administrated questionnaires during pregnancy and 6 month postpartum. Loss to follow-up is known to be higher among heavy smokers in this cohort [40], [41], and the prevalence of overweight is somewhat lower in the cohort children than in the general population, which may have induced underestimation of the smoking effects. Tobacco smoking, especially during pregnancy and lactation, is not well accepted in many societies. In addition, questions on postnatal smoking were presented along with questions on breastfeeding which may have lead to an underreporting of tobacco use, or in some cases possible misclassification [42], [43]. As questions of postpartum smoking in Interview 3 was asked in relation to breastfeeding we may have failed to include some mothers who smoked postpartum, but who reported no breastfeeding. Nevertheless, from the descriptive results (presented in Table 1) we see that at least three per cent of mothers reported smoking without breastfeeding. Moreover, a review and meta-analysis concluded that self-reports of smoking are accurate in most studies [44], [45]. The presence of such possible bias may have lowered the observed effect of the postpartum smoke exposure compared with the effect of smoke exposure during pregnancy.

The children were followed up through to age 7 years, and although the outcomes were based on self-reported information, it was concluded in an earlier validation study that the outcome data was valid with no random or systematic bias [31].

## Conclusions

Smoking both in the prenatal and early postnatal life, by otherwise healthy mothers increased term delivered childrens risk of overweight at age 7 years, independent of low BW, paternal smoking, and relevant confounders. Prenatal smoking exposure exhibited a clear dose-response relationship. Moreover, if mothers smoking continued into the child's early postnatal life the OR of overweight was significantly higher than for children exposed to smoke only during pregnancy. Findings emphasizes the need for prevention of any tobacco exposure of infants.
